# Pleiotropy of Progesterone Receptor Membrane Component 1 in Modulation of Cytochrome P450 Activity †

**DOI:** 10.3390/jox14020034

**Published:** 2024-05-01

**Authors:** Isabel S. Barata, José Rueff, Michel Kranendonk, Francisco Esteves

**Affiliations:** 1 Department of Pediatrics, Division of Endocrinology, Diabetology and Metabolism, University Children’s Hospital, University of Bern, 3010 Bern, Switzerland; isabel.sousabarata@unibe.ch; 2 Translational Hormone Research Program, Department of Biomedical Research, University of Bern, 3010 Bern, Switzerland; 3 Graduate School for Cellular and Biomedical Sciences, University of Bern, 3012 Bern, Switzerland; 4 ToxOmics, NOVA Medical School, Faculdade de Ciências Médicas, NMS|FCM, Universidade NOVA de Lisboa, Campo Mártires da Pátria 130, 1169-056 Lisboa, Portugal; jose.rueff@nms.unl.pt

**Keywords:** progesterone receptor membrane component 1 (PGRMC1), cytochrome P450 (CYP), drug metabolism, protein–protein interaction (PPI)

## Abstract

Progesterone receptor membrane component 1 (PGRMC1) is one of few proteins that have been recently described as direct modulators of the activity of human cytochrome P450 enzymes (CYP)s. These enzymes form a superfamily of membrane-bound hemoproteins that metabolize a wide variety of physiological, dietary, environmental, and pharmacological compounds. Modulation of CYP activity impacts the detoxification of xenobiotics as well as endogenous pathways such as steroid and fatty acid metabolism, thus playing a central role in homeostasis. This review is focused on nine main topics that include the most relevant aspects of past and current PGRMC1 research, focusing on its role in CYP-mediated drug metabolism. Firstly, a general overview of the main aspects of xenobiotic metabolism is presented (I), followed by an overview of the role of the CYP enzymatic complex (IIa), a section on human disorders associated with defects in CYP enzyme complex activity (IIb), and a brief account of cytochrome *b*_5_ (cyt *b*_5_)’s effect on CYP activity (IIc). Subsequently, we present a background overview of the history of the molecular characterization of PGRMC1 (III), regarding its structure, expression, and intracellular location (IIIa), and its heme-binding capability and dimerization (IIIb). The next section reflects the different effects PGRMC1 may have on CYP activity (IV), presenting a description of studies on the direct effects on CYP activity (IVa), and a summary of pathways in which PGRMC1’s involvement may indirectly affect CYP activity (IVb). The last section of the review is focused on the current challenges of research on the effect of PGRMC1 on CYP activity (V), presenting some future perspectives of research in the field (VI).

## 1. Introduction: Overview of Xenobiotic/Drug Metabolism

Exogenous compounds entering the human organism or naturally formed endogenous substances may undergo metabolic conversion (biotransformation). Regarding xenobiotics, this metabolism converts these compounds to more hydrophilic, water-soluble metabolites, facilitating their excretion [1,2,3]. Biotransformation reactions occur virtually in all tissues but, in terms of capacity, are mostly located in the liver. These reactions are traditionally grouped into two types, Phase I: oxidation, reduction, or hydrolysis of primarily lipophilic xenobiotics into more polar molecules; and Phase II: conjugation reactions with charged molecules [1,3,4,5]. The cytochrome P450 (CYP) enzyme family is central in biotransformation, comprising 70–80% of all Phase I xenobiotic metabolism [6,7]. CYPs are also involved in other key biochemical processes such as cholesterol, fatty acid, and eicosanoid homeostasis, steroidogenesis, or vitamin metabolism [1,7,8,9].

**Figure 1:**
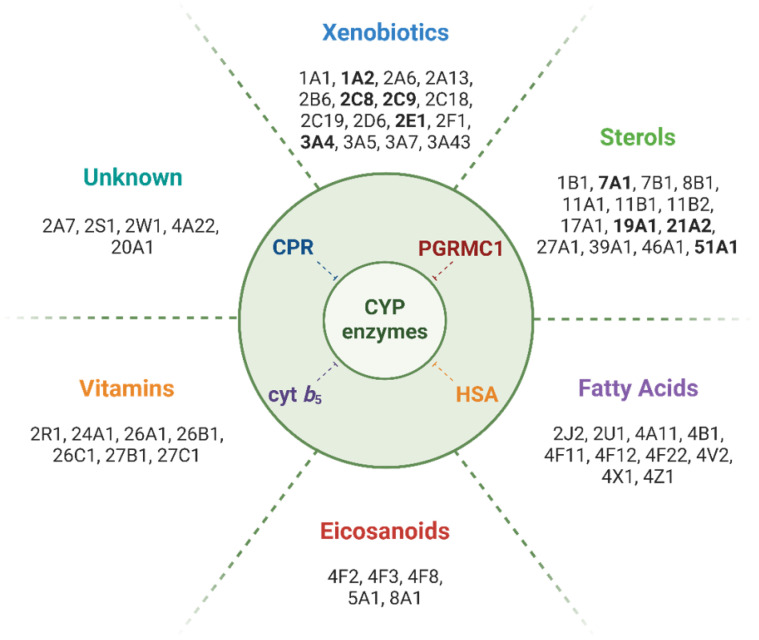
Main classes of compounds metabolized by CYPs and isoforms involved in their biotransformation. Human CYP isoforms for which binding or enzyme activity modulation by PGRMC1 has been reported are highlighted in bold. Note: To the best of our knowledge, no direct effect of PGRMC1 has been reported for CYP isoforms metabolizing fatty acids, eicosanoids, or vitamins, or for those whose substrate class is unknown.

Protein−protein interactions (PPI) play a critical role in CYP-mediated metabolism (reviewed in Kandel and Lampe, 2014). The interaction of CYPs with their redox partners, (i) adrenodoxin (or ferredoxin, FDX) for mitochondrial CYPs, (ii) cytochrome P450 oxidoreductase (CPR, encoded by the *POR* gene), mediating the donation of two electrons to microsomal CYPs during the catalytic cycle, and with (iii) cyt *b*_5_, an allosteric mediator that additionally may donate the second electron in CYP’s catalytic cycle, have been intensively studied [10,11,12,13,14,15,16,17,18]. Compared to the high versatility of PPI observed for microsomal CYPs and their redox partners (CPR and cyt *b*_5_), the ones of the mitochondrial CYP electron transport system are highly specific [19]. During the last two decades, other proteins potentially able to establish PPI with CYPs, and effecting their functions, have been identified. In addition to homo/heteromeric CYP−CYP interactions [20,21,22,23,24], proteins such as serum albumin [25,26,27,28] or progesterone receptor membrane component 1 (PGRMC1) can bind to CYPs, altering their activity (Figure 1) [29]. In particular, the interaction of CYPs with PGRMC1 has been shown to affect metabolic routes, including drug and xenobiotic biotransformation, but also steroids, fatty acids, cholesterol, and eicosanoids synthesis [30,31,32]. Although substantial progress has been made in elucidating the pathways through which PGRMC1 seems to contribute to variation in CYP-mediated responses, specific aspects of the underlying molecular mechanisms still require further clarification. In this brief review, we summarize the current status of research on PGRMC1:CYP interactions and PGRMC1’s pleiotropic effects on CYP activity.

## 2. The CYP Enzyme Complex

### 2.1. Constitution of the CYP Enzymatic Complex

CYPs are membrane-bound heme-thiolate monooxygenases initially found in animal liver microsomes [33,34]. Subsequently, their presence was not only uncovered in the mitochondria of steroidogenic organs of mammals but also in all eukaryotic organisms, plants, and fungi, and even in some prokaryotes [35]. The binding of carbon monoxide (CO) by the heme moiety in its reduced state gives rise to a unique 450 nm optical absorption peak, first described by Omura and Sato [36].

The Human Genome project uncovered 58 pseudogenes and 57 coding genes for human CYP proteins, which have been classified into 18 families and 44 subfamilies according to their amino acid sequence homology [8]. Human CYPs can also be classified on their subcellular location, either mitochondrial (seven of type I) or microsomal (50 of type II). Both types require electrons for their activity, originating from reduced nicotinamide adenine dinucleotide phosphate (NADPH), which must reach the heme iron of the CYP through an electron-transport chain that differs between the two types of CYP [37]. Microsomal CYPs are reduced by CPR, a membrane-bound diflavin reductase. Besides CYPs, CPR sustains the activity of non-CYP enzymes, including heme-oxygenase (HO), squalene monooxygenase, and cyt *b*_5_, albeit the latter is not exclusively reduced by CPR. Additionally, CPR may also directly reduce several small molecules, contributing directly to Phase I metabolism [11]. For mitochondrial CYPs, FDX mediates electron transfer after being reduced by adrenodoxin reductase (or ferredoxin reductase, FDXR) [38,39].

CYPs are present in almost all tissues but in higher concentrations in the small intestine, and particularly in the liver, the principal site of xenobiotic metabolism. CYPs mediate a wide range of different types of reactions, catalyzing the biotransformation of an immensity of compounds [40], in many cases with overlapping substrate specificity, i.e., a single compound may be metabolized by different CYP isoforms, through multiple reaction pathways yielding different metabolites. Additionally, the same compound, when metabolized by a single CYP, may yield different metabolites [1,2]. Moreover, substrate binding may modulate the interaction of CYP with its partner proteins, affecting both turnover rates and inducing deviated metabolite patterns [13,41,42].

In the liver, CYPs are involved in the metabolism of exogenous compounds (e.g., therapeutic or recreational drugs, pesticides, industrial chemicals, and food contaminants), but also in the metabolism of cholesterol, bile acids, and steroid hormones. In steroidogenic tissues, CYPs are involved in the metabolism of sex steroids (in gonads and placenta) and corticosteroids (in the adrenal cortex). Additionally, CYPs play important roles in cellular homeostasis, such as in the metabolism of vitamins and unsaturated fatty acids [1]. Isoforms from families 1, 2, and 3 are mostly involved in the metabolism of xenobiotics. CYP3A4 and CYP2D6, particularly, contribute to the biotransformation of most clinical drugs [43]. Most of the xenobiotic metabolizing reactions result in the detoxification of potentially harmful compounds. However, in some cases, these may result in the activation of compounds, i.e., producing metabolites with increased chemical reactivity (e.g., pro-carcinogens or carcinogenic metabolites), or of prodrugs to their pharmacologically active metabolites [1,2].

### 2.2. Metabolic Phenotypes and Pathologies

Disorders associated with alterations in the expression and activity of one or several of the protein factors of the CYP enzymatic complex may present broadly different clinical manifestations due to the wide range of pathways mediated by different CYP isoforms. Altered enzyme function can result in disrupted homeostasis of endogenous compounds, defective xenobiotic inactivation, or altered levels of prodrug activation [44]. Genetic variability of proteins of the CYP enzyme complex has been associated with multiple human diseases [9,45]. Polymorphisms of drug-metabolizing CYPs (especially of CYPs 2D6, 2C19, 2C9, 2B6, 3A5, and 2A6) or of their electron donor CPR may result in different metabolizer phenotypes (from ultrarapid metabolizers to poor metabolizers) and are associated with the occurrence of adverse drug reactions and a lack of drug efficacy [1,11,42,43].

Besides the clinical relevance of studying variations in CYP metabolism to assess different individual profiles of inactivation or activation of drugs and carcinogens, dysfunctions in CYP metabolism have been associated with a multitude of genetic pathological conditions. In this context, several studies demonstrated that mutations in three CYP genes (*CYP7A1*, *CYP7B1*, and *CYP27A1*) affect bile acid synthesis and cholesterol homeostasis, with different phenotypes leading to metabolic pathologies [46,47,48,49,50,51]. Defects in CYPs 2R1 and 27B1, involved in the conversion of vitamin D into its physiologically active form, are genetic causes of rickets [52,53,54,55]. Deficiency in the retinoic acid-metabolizing enzymes from the CYP26 family is associated with skeletal and craniofacial anomalies [56,57]. CYP1B1 dysfunction is associated with defects in eye development, causing eye-related disorders that frequently lead to the development of primary congenital glaucoma [58,59,60]. CYP4 enzymes, mediating the synthesis of the eicosanoid 20-hydroxyeicosatetraenoic acid (20-HETE), have been associated with hypertension, salt and water balance, and kidney injury [61,62,63,64]. CYPs 5A1 and 8A1 (designated as thromboxane-A synthase (TBXAS1) and prostacyclin synthase (PTGIS), respectively), participate in prostanoid signaling, as part of the arachidonic acid metabolism pathway. Mutations in *TBXAS1* are causal to Ghosal hematodiaphyseal dysplasia syndrome, a condition characterized by increased bone density, anemia, and inflammation [65]. A splicing mutation of *PTGIS* was associated with hypertension [66].

Deficiency in CYPs involved in steroidogenesis—mitochondrial CYPs 11A1, 11B1, and 11B2, and microsomal CYPs 17A1, 19A1 (aromatase), and 21A2—leads to a broad spectrum of phenotypes involved in retained adrenal function and sex development disorders, hypertension, and skeletal abnormalities [67,68,69,70,71]. Mutations in cyt *b*_5_, FDX, and FDXR have also been identified in rare steroidogenesis disorders [37]. Moreover, cyt *b*_5_ missense mutations may cause methemoglobinemia [72,73,74].

Regarding CPR’s central functions, knockout (KO) of the *POR* gene (encoding CPR) is embryonically lethal in mice [75,76], while liver-specific *POR*^KO^ mice developed normally and were able to breed, albeit demonstrating compromised drug metabolism and bile acid production, and increased hepatic lipid levels [77]. These hepatic CPR-null mice presented deregulated expression of more than two hundred genes crucial to hepatic homeostasis, including increased expression of hepatic drug-metabolizing CYPs and heme-oxygenase 1 (HO-1) [78,79]. *POR* deficiency can manifest in a broad spectrum that includes abnormal steroidogenesis (and consequent genital ambiguity and some cases of maternal virilization during pregnancy due to fetoplacental aromatase deficiency), Antley–Bixler syndrome (characterized by skeletal abnormalities) [11,37,80], compromised drug metabolism [81,82], and altered heme metabolism through its role in HO-1 activity [83,84].

Many CYP isoforms have been associated with different types of cancer and correlated with adverse drug reactions and treatment failure [85]. Polymorphisms and altered expression of CYPs mediating vitamin D metabolism, androgen and estrogen metabolism, and drug biotransformation could function as biomarkers of cancer susceptibility and drug treatment efficacy in cancer patients [86,87,88,89,90,91]. Many of the CYPs long considered orphan CYPs (e.g., CYP2U1, 2S1, and 4V2) have been implicated in cancer and several other diseases [92,93,94,95,96,97,98,99]. Major drug-metabolizing CYPs, such as 1A2, 2D6, 2C9, and 3A4, are implicated in detrimental effects by bioactivation of substances to toxic metabolites [100], drug–drug or herb–drug interactions [101,102,103], and chemoresistance [104,105]. This extensive impact urges a comprehensive assessment of all manners of CYP activity modulation, not only of expression regulation, the influence of non-synonymous mutations, or direct inhibition by chemical compounds, but additionally those of PPIs that may modulate CYP activity.

### 2.3. Cyt b_5_’s Modulatory Effect on CYP Metabolism

Cyt *b*_5_ was the first protein, besides the canonical redox partners FDX and CPR, discovered to modulate CYP activity [106,107]. This small (approximately 15 kDa) hemoprotein is involved in several biochemical processes. It is present in three different isoforms in mammals: membrane-bound to the endoplasmic reticulum (ER), as an outer mitochondrial membrane-bound protein, and in a soluble cytosolic form. The ER-associated cyt *b*_5_ has been shown to be involved in CYP-catalyzed reactions, in a CYP isoform- and substrate-dependent manner [11,108,109]. Cyt *b*_5_ can be involved in CYP metabolism, as demonstrated in vitro and in vivo [110,111], by donating the second electron in the catalytic cycle, or by allosteric modulation, or even both, in particular for reactions mediated by CYP1A2, 2D6, 2E1, 3A4, 11A1, and 17A1 [108]. Hepatic microsomal cyt *b*_5_ null (HBN) mice presented significant changes in in vivo pharmacokinetics of both intravenously and orally administered drugs, and respective liver microsomes demonstrated affected in vitro rates of CYP-mediated metabolism of both model substrates and probe drugs [110]. Moreover, in the case of two specific CYP3A probe substrates (midazolam and metoprolol), cyt *b*_5_’s modulatory effect showed specificity for the site of oxidation, promoting the formation of one metabolite over the other, suggesting an allosteric effect [110]. In a later study, the same group showed that, for the metabolism of probe substrates by hepatic microsomes obtained by crossing the HBN mice (lacking hepatic cyt *b*_5_) with CYP2D6 or CYP3A4 humanized mice, cyt *b*_5_ had a substrate-specific effect, altering CYP2D6 and CYP3A4-mediated metabolism, shown both in vitro and in vivo [111]. The addition of exogenous cyt *b*_5_ rescued the CYP activity of microsomal fractions and stimulated the activity of *E. coli* membranes containing recombinant CPR and CYP2D6, confirming the direct effect of cyt *b*_5_ on CYP-mediated drug metabolism.

Cyt *b*_5_’s effect on steroidogenesis and drug metabolism has been a relevant study subject [17,107,112,113]. For CYP17A1, which has both a 17α-hydroxylase and a 17,20-lyase activity, cyt *b*_5_ has been demonstrated to favor the lyase reaction in steroidogenic tissues and the resulting synthesis of sex steroids over glucocorticoids, via allosteric stimulation of CYP17A1 seemingly without electron transfer [108,109]. Furthermore, the affinity of CYP17A1 for cyt *b*_5_ seems to be augmented in the presence of steroidal substrates [41], and cyt *b*_5_ may attenuate detrimental effects of structural mutations of CYPs, as it apparently affects CYP1A2 naturally occurring variants to behave more like the wild-type, when co-expressed using a bacterial cell model [18].

The potential of other proteins besides CPR to modulate CYP enzymatic activity broadens the field of study of disorders in steroidogenesis and xenobiotic metabolism and highlights the importance of in-depth characterization of genetic and non-genetic variability within the human population in the quest for understanding variability in drug response and therapeutic failure. The findings that the interaction of CYPs with other proteins such as cyt *b*_5_ can alter the pharmacokinetics of CYP reactions add further complexity to the study of detoxification pathways and prediction of therapeutic responses to drugs. PGRMC1, a protein that contains a cyt *b*_5_-like domain, can additionally play a role in CYP-mediated pathways, remaining, however, intriguing regarding its interaction and effect in the modulation of CYP enzymatic activity and consequently in drug pharmacokinetics. Owing to its resemblance to cyt *b*_5_, PGRMC1 has been hypothesized as a putative CYP modulator. Nevertheless, the evidence supporting its direct involvement in CYP activity is limited and inconclusive, with ambiguity compounded by contradictory findings in various reports.

## 3. Molecular Characterization of Progesterone Receptor Membrane Component 1 (PGRMC1)

### 3.1. PGRMC1 Expression, Intracellular Location, and Structure

PGRMC1 is a member of the membrane-associated progesterone receptor family, a group of four partially homologous proteins that share a similar cyt *b*_5_-like heme-binding region [114,115]. PGRMC1 and its paralog PGRMC2 (encoded by genes 300,435 and 607,735, respectively, of the OMIM database) have been described to be involved in the regulation of female reproductive functions and to act as receptors for progesterone [30,31,32,116]. They have been implicated in several biological processes, namely, mitosis by stabilizing the mitotic spindle and entry into the cell cycle, apoptosis, and cell migration. Disruption of the expression of both proteins has been linked to specific cancers, particularly ovarian and breast cancers, since both proteins are overexpressed in ovarian and breast tumor cells [117,118]. Although the evolutionary history of PGRMC1 and PGRMC2 is rooted in shared ancestry, divergent events led to the distinct genomic architectures observed today. Detailed analysis of the nucleotide sequences reveals significant variation between PGRMC1 and PGRMC2 [114,119]. Effectively, PGRMC1 and PGRMC2 exhibit distinct genomic organization, structural characteristics, tissue expression patterns, and roles in cellular processes. Moreover, PGRMC2 functions and modes of action, tissue-specificity, subcellular locations, and interaction network are less well characterized compared to PGRMC1, and limited information is available on its specific role in health and disease states [120,121]. As such, this review focuses on the study of PGRMC1 and on the mechanisms by which this specific protein is able to influence cytochrome P450-mediated metabolism.

**Figure 2:**
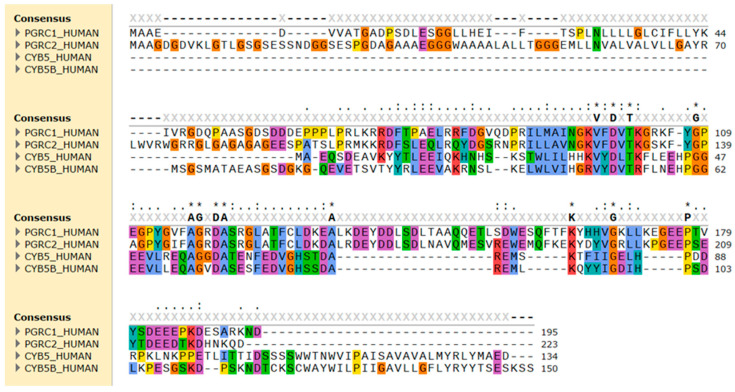
Sequence alignment of the amino acid sequence of human PGRMC1 (PGRC1), PGRMC2 (PGRC2), cytochrome *b*_5_ type A (CYB5), and type B (CYB5B). The alignment was performed using Clustal Omega and amino acid residues were colored by properties + conservation (Clustal X) in the SnapGene 7.0.3 software. Conserved hydrophobic amino acids are in blue, positively charged in red, negatively charged in magenta, polar in green, glycine in orange, and aromatic amino acids in cyan.

PGRMC1 was first discovered in rat and porcine liver by two independent groups: (i) as a rat DNA fragment differentially expressed following dioxin exposure [122], and (ii) as two protein fragments (suggested to be monomers and dimers of the same protein) with progesterone-binding activity, purified and partially sequenced from porcine liver [123]. Soon after, its encoding gene was cloned as the first human putative steroid membrane receptor, alongside its paralog PGRMC2 [116]. Subsequently, it was found in the inner zones of the rat adrenal cortex (leading to it being also termed an inner zone antigen) and suggested to play a role in steroid hormone synthesis and/or metabolism based on its tissue distribution [124]. PGRMC1 is highly homologous to PGRMC2, with sequences that diverge more distinctly at the N-terminal end and transmembrane domain (Figure 2), and both may have diverged from a common ancestor gene [120]. Although receiving less attention in research than its paralog, PGRMC2 has been implicated in fertility, heme chaperoning, adipocyte differentiation, and glucose homeostasis [116,125,126,127]. Regarding a possible effect on CYP activity, PGRMC2 has been shown to stably interact with CYP3A4 and CYP21A2 in vitro [128], and a *PGRMC2* single-nucleotide polymorphism (SNP) was associated with a CYP3A4 phenotype of decreased activity [129].

Expression of PGRMC1, cyt *b*_5_, and CYP11B1 (a mitochondrial CYP involved in the synthesis of glucocorticoids and mineralocorticoids) in cervical cancer cells (HeLa) showed PGRMC1’s intracellular location consistent with that of cyt *b*_5_, but not of CYP11B1, suggesting that PGRMC1 is associated with the ER membrane [130]. In other studies, PGRMC1 was also localized to the mitochondria of rat adrenal tissue [124] and was suggested to be associated with the mitochondrial membrane [131]. PGRMC1 was perinuclearly localized in breast cancer cells [132], while in the nuclei or concentrated at or near the plasma membrane of granulosa cells [133]. PGRMC1 was detected in the ER network and nuclear membranes of human embryonic kidney and liver cancer cells (HEK293 and HepG2 cells, respectively), and it was also present in the plasma membrane of HepG2 cells [134], rat glial cells, and primary astrocytes [135]. PGRMC1 was shown to be present as a monomer in the cytoplasm and as a dithiothreitol-resistant PGRMC1 dimer in the nucleus of spontaneously immortalized granulosa cells [136]. These different reports suggest that the intracellular location of PGRMC1 may to some extent be cell-type specific, as previously suggested [137], and seem to indicate different cellular functions.

**Figure 3:**
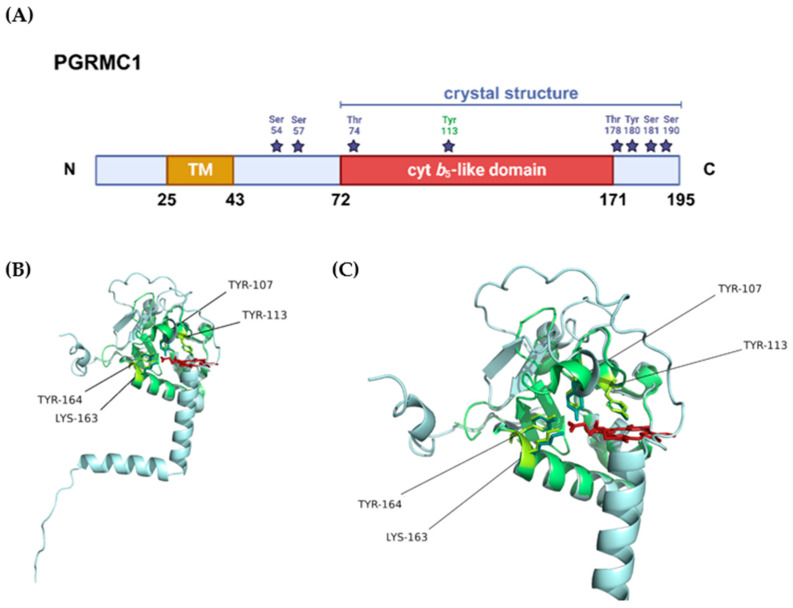
Structural representation of PGRMC1. (**A**) Protein domains and post-translational modifications (PTMs) according to the information provided in PGRMC1’s entry in the UniProt database (accession number: O00264). The blue bar represents the sequence length covered by the only crystal structure available for PGRMC1 (PDB 4 × 8Y), the purple stars represent the residues that can be phosphorylated, and highlighted in green is tyrosine 113, the residue that chelates the heme iron. (**B**) Superposition of the crystal structure available for PGRMC1 (PDB 4 × 8Y, in green) with the tridimensional structure predicted by AlphaFold [152,153] (in blue). The amino acid residues that seemingly interact with heme are highlighted in the tridimensional structure in yellow and represented as sticks, the heme cofactor is represented in red. (**C**) Closer view of the heme-binding pocket with highlighted heme-interacting residues.

The *PGRMC1* gene, located on chromosome X (Xq24), encodes a 195 amino acid peptide with a short N-terminal luminal domain, a single membrane-spanning domain [116], and a long cytoplasmic domain containing the heme-binding region, binding motifs for SH2- and SH3-domain proteins, immunoreceptor tyrosine-based activation motifs (ITAMs), and several potential kinase binding sites [138]. The theoretical molecular weight predicted for PGRMC1 is 21 kDa but higher molecular weight forms, ranging from around 25 kDa to above 100 kDa, have been reported in several studies [30,130,136,139,140]. The 70 and 100 kDa apparent molecular weight forms of PGRMC1 were predominantly associated with the ER/mitochondria and nuclear fractions, respectively, in HEK293 cells, and the higher molecular masses were explained by posttranslational modifications (PTMs) (see Figure 3A) [141]. Phosphorylation, acetylation, ubiquitination, and sumoylation of native PGRMC1 seem to regulate the interaction of PGRMC1 with other proteins, and manipulation of its phosphorylation status led to alterations in cell metabolism, shape and motility, genomic stability, and CpG methylation [142,143].

### 3.2. Heme-Binding and Dimerization in PGRMC1’s Function

Reports that homologs of PGRMC1 bind heme have long existed [144,145,146], especially when taking into account its cyt *b*_5_-like domain (Figure 3). Min et al. reported that PGRMC1 binds a high-spin *b* type heme chromophore, possibly in its monomeric, dimeric, and trimeric forms [130]. The group generated PGRMC1 mutants by site-directed mutagenesis of amino-acid residues containing imidazole, thiol, and phenol as side-chain groups which potentially could bind heme, with a special focus on three tyrosines and a cysteine, present in the putative heme-binding region/pocket. A Tyr107Phe/Tyr113Phe-PGRMC1 double mutant and one in which four consecutive amino acid stretches (Asp99-Lys102, containing three amino acid residues conserved in PGRMC1 and cyt *b*_5_) were mutated presented significantly lower heme-binding capacity, suggesting the potential involvement of these residues in heme-binding. Another in vitro study revealed that the created Asp120Gly PGRMC1 mutant did not bind heme and caused increased susceptibility of breast cancer cells to doxorubicin and camptothecin, two topoisomerase inhibitors used as chemotherapeutic agents [147]. This heme-binding defective mutant additionally showed reduced progesterone responsiveness [139], and was later implicated in the inhibition of MDA-MB-468 breast cancer cell proliferation, as opposed to non-mutated MDA-MB-468 cells, for which progesterone was shown to promote cell growth [148].

Earlier studies reported noncovalent binding of the yeast homolog, Dap1p (damage resistance protein 1), and PGRMC1 to high-spin, ferric heme—although with only maximal heme loading of 30% [144]. In this binding, heme was five-coordinated, with tyrosine as the axial ligand, in contrast to the six-coordinated, low-spin ferric heme in cyt *b*_5_ [130,144,149,150]. Human PGRMC1 and yeast Dap1p bind heme in both oxidation states, but with higher apparent affinity for the ferric form [149,150]. Clarification of the tyrosine coordinating residue in heme binding has been the subject of several studies over the years. Tyr164 was suggested as the axial ligand, considering its conservation in all membrane-associated progesterone receptor family members [144]. However, the X-ray crystal structure of the PGRMC1 cytosolic domain revealed a five-coordination of the iron by the axial Tyr113 residue [151]. Three other amino acid residues, Tyr107, Lys163, and Tyr164 (Figure 3B,C), seem to coordinate heme in the binding pocket through hydrogen-binding via heme’s propionate groups [151], confirming the previously predicted importance of tyrosines Tyr107, Tyr113 [130], and Tyr164 [144] for heme-binding. The open surface of the heme-binding pocket was suggested to mediate in vitro dimerization, with the protruding heme moieties in each monomer interacting by hydrophobic “heme–heme” stacking. This in vitro dimerization was shown to be inhibited by CO, leading to the dissociation of the dimer. In addition, the Tyr113Phe mutant seemingly does not bind heme (the addition of heme to apo-PGRMC1 mutant did not lead to increased molecular size) and is not able to interact in vitro with other proteins known to bind to PGRMC1 [151]. As such, dimerization was deemed necessary for the interaction of PGRMC1 with protein partners as shown for CYPs (1A2 and 3A4) and the epidermal growth factor receptor (EGFR), involved in signal transduction during cancer proliferation. Additionally, it has been previously suggested that the PGRMC1 dimer is predominantly found within the nucleus, where it may be involved in gene expression regulation [136]. Dimerization of PGRMC1 could also be a regulatory feature, a mechanism in which the cell modulates PGRMC1’s activity not only by PTMs, as mentioned above, but also by regulating dimerization and thus affecting PGRMC1’s ability to interact with its binding partners.

## 4. PGRMC1 Pleiotropic Effects on CYP Activity

### 4.1. Evidence of Direct Effects of PGRMC1 on CYP Metabolism

Despite its name linking PGRMC1 (and 2) to progesterone signaling, this protein shares more homology with cyt *b*_5_-related proteins rather than hormone receptors [154,155]. Due to the similarity to cyt *b*_5_, PGRMC1’s role as a CYP modulator has been questioned. However, studies implicating its direct role in CYP activity remain somewhat scarce and relatively inconclusive, with conflicting reports adding to this uncertainty. PGRMC1 and other membrane-associated progesterone receptor proteins were suggested to be evolutionarily linked to CYP activity and to have co-evolved with specific CYPs and some mitochondrial proteins. The most closely co-evolving genes were considered to be CYP1A1, 2A13, 2A7, 2C18, 2C19, 2C9, 2J2, 2R1, 2W1, and TBXAS1, and, besides mitochondrial CYPs, cyt *b*_5_ reductase and solute carrier family 22, among others [156]. The microsomal CYP1A1, 2A13, 2C9, 2C18, and 2C19 are mostly associated with xenobiotic metabolism, while CYP2J2 and 2R1 have fatty acids and vitamin metabolism, respectively [43]. CYP2A7 and the mitochondrial CYP2W1 are often termed “orphan” CYPs [157], as the classes of compounds metabolized by these are not yet fully characterized.

The first link between PGRMC1 and CYP activity can be traced back to 1988, when antibodies directed towards the rat homolog were reported to decrease CYP21A2 and 11B1-specific activities [158], suggesting a functional role of PGRMC1 in steroid metabolism. Subsequent studies investigated the effect of CYP21A2-mediated steroid 21-hydroxylation reaction in COS-7 cell expression systems, and results suggested that rat PGRMC1 enhanced CYP21A2 activity. However, when using a microsomal CYP electron-transport reconstitution system, 21-hydroxylation of progesterone seemed to be inhibited by higher PGRMC1 levels [130], which might indicate stoichiometry issues. The activity of CYP17A1 was also assessed to clarify if PGRMC1 could influence this isoform’s activity in a similar manner as exhibited by cyt *b*_5_. In the CYP17A1-reconstitution system, cyt *b*_5_ had no significant influence on the 17-hydroxylation of progesterone but activated the lyase reaction of 17α-hydroxyprogesterone, however, rat PGRMC1 did not show this effect. The co-expression of specific PGRMC1 mutants with CYP21A2 in COS-7 cell expression systems by Min et al. showed modulation of CYP activity, i.e., when compared to a null vector, wild-type PGRMC1 co-expression increased CYP21A2 hydroxylation of progesterone by about two-fold, the double mutant Tyr107Phe/Tyr113Phe increased activity by approximately 60%, while the Asp99-Lys102 mutant decreased activity by nearly 75%. Protein levels showed higher contents of the Asp99-Lys102-mutated PGRMC1 along with lower CYP21 protein levels when compared to wild-type PGRMC1-expressing cells. These lower CYP21 contents were confirmed as not due to repressed expression of CYP21 by the higher mutant PGRMC1 expression, but rather attributed to post-translational events. Considering this, the authors suggested a role for PGRMC1 as a heme-supplier for CYP-mediated reactions [130]. Heme transfer between CYP and cyt *b*_5_ has been previously reported [159]. The homology of PGRMC1 with cyt *b*_5_ (Figure 2) might indicate a similar function.

Until Espenshade and colleagues showed that the yeast PGRMC1 homolog Dap1 binds and positively regulates two fungal CYP isoforms (Erg5 and Erg11, required for sterol biosynthesis in yeast), cyt *b*_5_ and CPR were the only proteins known to interact with and affect the activity of microsomal CYPs. The study reported that Dap1 was required for lanosterol metabolism, mediated by Erg11, homolog to human CYP51A1, and responsible for the synthesis of the yeast cholesterol equivalent, ergosterol. The same authors evidenced that human PGRMC1 binds CYP7A1, 21A2, and 51A1, all involved in sterol metabolism, and 3A4, specifically involved in xenobiotic metabolism and clearance of a majority of known drugs [160]. The binding of PGRMC1 to rabbit CYP2C2 and human CYP2C8 and CYP3A4 (all drug-metabolizing CYPs) was later reported with immunoprecipitation assays with lysates of HEK293 cells, co-expressing PGRMC1 and each CYP isoform [134]. Moreover, by co-expressing PGRMC1 with chimeras containing the N-terminal domain of CYP2C2 or its cytosolic domain, the authors reported that PGRMC1 bound much more efficiently to CYP2C2’s cytosolic domain. Inhibition of enzymatic activity of CYP2C2, 2C8, and 3A4 was described in HEK293 and HepG2 cells which were stably expressing either non-silencing control or PGRMC1 siRNA with tagged-PGRMC1. CYP51 activity was increased by PGRMC1 in HEK293 cells, in agreement with the data from Espenshade’s group. This different modulation of CYP51A1 and CYPs 2C2, 2C8, and 3A4 activities suggested an isoform-dependent effect for PGRMC1, resembling the effects of cyt *b*_5_. Induced overexpression of CPR in HEK293 cells increased CYP activity of drug-metabolizing CYPs (2C2, 2C8, and 3A4) while having little effect in HepG2 cells, which express much higher levels of endogenous CPR compared to HEK293 cells. The overexpression of CPR in HEK293 cells seemed to revert, to some extent, the inhibition of CYP activity by tagged PGRMC1 overexpression. The same report described coimmunoprecipitation of PGRMC1 with CPR, suggesting strong interactions between these proteins, and that the simultaneous co-expression of CYP2C2, CPR, and PGRMC1 decreased CPR-PGRMC1 binding while having little effect on the binding of CYP2C2 to PGRMC1 [134].

Using co-expression systems for PGRMC1 and CYPs in HepG2 cells, Oda et al. demonstrated that higher levels of PGRMC1 diminished CYP3A4 and CYP2C9 enzyme activities for different substrates, while having a less pronounced effect on CYP2E1 activity. The authors observed that PGRMC1 overexpression modulated CYP activities by increasing the *K*_m_ and decreasing the *k*_cat_ of the CYP3A4-mediated reactions, while decreasing the *k*_cat_ of the CYP2C9-mediated reactions [137], although stoichiometry discrepancies between redox partners in this study might have had influenced outcomes. The overexpression of PGRMC1 (by 5.6-fold at protein level) in human hepatocytes corroborated the inhibitory effect on CYP activity observed using the HepG2 cells co-expression systems, while coimmunoprecipitation suggested direct binding of PGRMC1 to the three CYP isoforms assessed, although with different affinities. This study also reported that CPR and PGRMC1 coimmunoprecipitation was not observed in HEK293 transiently expressing FLAG-PGRMC1 and Myc-CYP, an outcome the authors attributed as a result of low expression of CPR.

In order to examine the effect of PGRMC1 on aromatase (CYP19A1) activity in breast cancer, MCF-7 (hormone-responsive) cells were engineered for expression of an epitope-tagged aromatase and PGRMC1 was knocked down [161]. Compared with control cells, the knockdown cells presented reduced aromatase activity without changes in aromatase protein level, indicating functional stimulation of PGRMC1 on CYP19A1 activity. This seems to be confirmed in the same study when increased aromatase activity was detected when incorporating purified recombinant PGRMC1 with CYP19A1 “Supersomes”. A more recent study, by the laboratory of Espenshade, identified 32 binding partners for Flag-PGRMC1 from liver membrane fractions of *Pgrmc1* whole-body KO mice expressing Flag-tagged full-length PGRMC1, using mass spectrometry [162]. Thirteen (41%) of the binding partners were CYPs, while CPR was not among the identified proteins. Binding to mouse CYP1A2, 2E1, 3A, and 51A was validated by Western blotting. The *Pgrmc1* KO mice presented lower contents of CYP proteins, which were not related with reduced CYP transcription as some were even upregulated. PGRMC1 seemed to stabilize CYP1A2 post-translationally, increasing its half-life by 67%, and that of CYP2E1 protein levels by 20%. Importantly, *Pgrmc1* KO mice were protected from acetaminophen-induced toxicity (mainly mediated by CYP2E1 metabolism), demonstrating that PGRMC1 has an in vivo effect on CYP2E1 activity. The same study also reported that the axial iron-coordinating residue (Tyr113) was not required to maintain CYP levels—no significant differential expression was observed between liver microsomes derived from *Pgrmc1* KO mice expressing either human wild-type or the Tyr113Phe PGRMC1 mutant—or in supporting their enzymatic activity. Contradictory results have been reported regarding Tyr113 and its role in heme binding and CYP activity. Kabe et al. described a Tyr113Phe PGRMC1 mutant lacking the N-terminal segment, which did not bind either CYP1A2 or CYP3A4, and concluded this was due to the lack of heme of this mutant [151]. However, McGuire et al. reported heme binding of a truncated human Tyr113Phe mutant and interaction of the full-length Flag-tagged mutant with 18 CYP isoforms (five more than those binding to the Flag-tagged wild-type). This study advocated that mutation of Tyr113 alone did not prevent heme-binding—a conclusion also presented by an earlier study [130]—and that additional mutations of Lys163 and Tyr164 residues were needed to observe this specific lack of binding [162]. This triple mutant (Tyr113Phe/Lys163Ala/Tyr164Phe) was able to bind CYP1A2 in *PGRMC1* KO human fibroblast cells and *Pgrmc1* KO mouse liver, suggesting that PGRMC1 binding to CYPs occurs independently of the presence of heme. However, whether this triple PGRMC1 mutant stimulated CYP1A2 enzymatic activity remained unclear [162]. This ability to interact with CYP1A2 despite the detrimental effect of the mutations on specific heme-binding ability reinforces the hypothesis that PGRMC1’s effect on CYP activity has, similarly to that of cyt *b*_5_, an allosteric modulatory component, independent of heme binding. Conversely, the observed decreased CYP21A2 protein levels in the presence of the heme-binding defective PGRMC1 mutant (four consecutive amino-acid stretch) assessed in Min et al.’s study [130], seems to contradict this independence of heme binding in the effect of protein stabilization.

A truncating deletion of PGRMC1 was found in the etiology of developmental cataracts, rationalized by a potential disruption of PGRMC1’s interaction with CYP51A1 and consequently altered cholesterol biosynthesis [163]. In addition, a His165Arg mutation (located in the cyt *b*_5_ domain of PGRMC1), identified in a heterozygous patient with premature ovarian failure, was shown to interfere with PGRMC1-mediated progesterone’s anti-apoptotic action and abolish binding to CYP7A1, important for the synthesis of bile acid and the regulation of cholesterol levels [164]. This study also sought to assess the binding of this missense mutant to CYP3A4 and CYP21A2, but endogenous expression of these CYPs was too low in HEK293 cells for such evaluation. The incapacity of the His165Arg mutant to bind CYP7A1 may be a result of an impediment in heme-coordination by the adjacent Tyr164 amino acid residue [164], or of minor structural deviations with implication in the interaction and allosteric effect of this PGRMC1 mutant protein.

In conclusion, this section summarizes reports of direct binding of PGRMC1 to several CYPs, and the isoform-dependent modulatory effects of PGRMC1 on CYP metabolic activity. Most importantly, human PGRMC1 was reported to modulate human CYP isoforms of the three families responsible for the majority of hepatic xenobiotic metabolism: In vitro, PGRMC1 binds to CYP1A2, 2C8, and 3A4 and modulates the enzymatic activity of 2C9, 2E1, and 3A4 (see Figure 1) [134,137,160,162]. Moreover, PGRMC1 modulates the activity of CYP isoforms involved in sterol metabolism, as reported for CYP7A1, 19A1, 21A2, and 51A1 [130,134,160,161,164].

### 4.2. PGRMC1’s Involvement in Pathways with Potential Indirect Effects on CYP Metabolism

PGRMC1 has been credited with roles in an immensity of pathways, including progesterone signaling and interaction with CYPs. The myriad of functions attributed to this protein may be mediated through differential interactions with known binding partners and/or differential PTM status. Additionally, the role of PGRMC1 as a signaling and metabolic hub [30] may add another layer of complexity to CYP activity modulation as, in the absence of a direct PGRMC1:CYP interaction, there may still be an indirect effect of PGRMC1 on CYP expression and activity. This seems to occur through other CYP-independent pathways in which PGRMC1 is involved and that are summarized in this section.

PGMRC1 is assumed to bind not only other non-CYP complex proteins, heme, and progesterone, but also testosterone, glucocorticoids, and other cholesterol metabolites, albeit with lower binding affinities [165]. The discovery of extranuclear steroid hormone receptors brought a new understanding of hormone-induced rapid signaling that triggers downstream pathways, able to mediate responses independently of the traditional known effect of hormones as genomic regulators [166,167]. PGRMC1 is generally thought to bind progesterone at the heme/ligand-binding cleft contained within the cyt *b*_5_-like domain [149], although studies with deletion mutants suggested that the progesterone binding site is localized in the transmembrane domain and an adjacent segment of the C terminus [139]. Moreover, there is some debate about whether the involvement of PGRMC1 in progesterone signaling entails direct or indirect actions, resulting from directly binding progesterone or from interaction with other progesterone-binding proteins [167]. In vitro studies showed that PGRMC1 mediates the anti-apoptotic action of progesterone, interacting with the plasminogen activator inhibitor RNA-binding protein 1 (PAIRBP1, also known as SERBP1) [168], independently of the classical nuclear progesterone receptors (PR) [132]. However, this interaction does not strictly require progesterone binding, although it may couple PGRMC1 to downstream components of the progesterone-PGRMC1 signal transduction pathway [168,169]. Other studies suggested that the expression-regulating component to PGRMC1’s anti-apoptotic mechanism of action requires the presence of the PGRMC1 dimer and that progesterone activation of PGRMC1 modulates the expression of regulators of mitochondrial outer membrane permeabilization [136]. The progesterone-PGRMC1 signaling pathway and the sumoylation status of PGRMC1 also seem to modulate apoptosis by regulating the activity of the transcription factor Tcf/Lef [140], which is activated through the Wnt/β-catenin pathway. This mechanism has been shown to modulate CYP expression [170,171,172,173], interplaying with aryl hydrocarbon receptor (AhR) and constitutive androstane receptor (CAR) activation, both important trans-factors in the regulation of CYP expression [174,175].

PGRMC1 has also been implicated in resistance to the EGFR inhibitor erlotinib by binding to EGFR and initiating crosstalk between the Wnt/β-catenin and NF-κB pathways [176]. Another report suggested that PGRMC1 suppressed the p53 and Wnt/β-catenin pathways to promote self-renewal and inhibit early differentiation in human pluripotent stem cells [177]. The association between PGRMC1 and EGFR was linked with NF-κB activity stimulation in a murine model of hepatocellular carcinoma, resulting in increased proinflammatory interleukin-6 (IL-6) production. These effects were modulated by exposure to erlotinib or by PGRMC1 loss with a resultant decrease in EGFR levels [178]. PGRMC1 was also found to regulate TNF-α expression and, consequently, TNF-α-induced expression of genes involved in neuroinflammation [179], and to partially mediate the anti-inflammatory effects of medroxyprogesterone acetate, which inhibits TNF-α [180]. The involvement of PGRMC1 in inflammatory pathways and in regulating the production of proinflammatory factors such as IL-6, NF-κB, and TNF-α, modulators of CYP expression [85,181,182], may warrant PGRMC1’s indirect role in CYP expression regulation.

Besides the putative role as a hormone receptor, the fact that PGRMC1 is broadly expressed in most tissues suggests both progesterone-dependent and progesterone-independent functions [32]. PGRMC1 overexpression was found in various types of cancer [183,184,185,186], associated with poor prognosis and aggressiveness, and its overexpression was proposed as a valuable prognosis biomarker [187,188,189]. A differential PGRMC1 phosphorylation status was shown between estrogen receptor-positive (ER+) and negative (ER-) breast cancers (BC) [190], an indication that PGRMC1 may be involved in the clinical differences between tumors with distinct ER statuses, in a phosphorylation-dependent manner. Overexpression of human PGRMC1 in BC cell lines concomitantly occurred with increased levels of membrane progesterone receptor α (mPRα) and estrogen receptor β (ERβ) [169]. Additionally, these cell lines demonstrated increased levels of estradiol and augmented expression of ERα and ERα-target genes, as well as upregulated EGFR activation and downstream signaling [191]. Interestingly, the downregulation of ERα decreased the expression of PGRMC1, leading to no repercussions on classical PR expression, while silencing of PGRMC1 led to a significant decrease in ERα expression [192]. Treatment of hormone receptor-positive BC cell lines with proliferation-promoting progestins increased Ser181-phosphorylation of PGRMC1 and its interaction with prohibitins, correlating with decreased binding of prohibitins to ERα and subsequent ERα activation, enabling the transcription of ERα-dependent genes and increasing proliferation [193]. CYP enzymes are involved in the steroidogenic pathway not only in sex steroid biosynthesis but also in their metabolism and inactivation [194,195]. An antagonistic estrogen-retinoic acid transcriptional signaling crosstalk described in neurons may affect CYP expression [196], as well as crosstalk with AhR, which has been reported as pro-estrogenic in some contexts by upregulating aromatase (CYP19A1) and contributing to ERα and ERβ transcriptional activity [197,198].

It should also be noted that steroid hormones may directly affect CYP expression and activity, based on the evidence that these types of hormones activate receptors that are in turn involved in CYP expression regulation, such as the CAR, the pregnane X receptor (PXR) and the AhR [1,199,200]. PXR mediates the genomic effects of several steroid hormones, including progesterone, and regulates the expression of several genes involved in the metabolism and elimination of potentially toxic substrates [201]. Estradiol activates both CAR and ER that synergistically induce CYP2B6 expression [202,203], and enhances CYP2A6 [203,204], CYP1B1 [205], and CYP3A4 expression and CYP2C9 and CYP2E1 enzyme activities without affecting their mRNA expression levels. In addition, progesterone has been shown to induce CYP2A6, CYP2B6, CYP2C8, CYP3A4, and CYP3A5 expression [203]. The involvement of PGRMC1 in ERα activation, previously described in this section, and the recognized crosstalk between the AhR and the ERα signaling pathways [206,207] may also translate to the effects of PGRMC1 on CYP expression. This interaction between steroid hormone signaling and xenobiotic metabolism regulation is particularly important in states of hormonal fluctuations and disturbance, such as pregnancy and certain types of cancer, leading to alterations in hepatic drug metabolism due to altered levels of drug-metabolizing enzymes. Moreover, a high inhibition of CYP21A2 (earlier described following inhibition of rat PGRMC1 [158]) could affect cortisol levels. As cortisol is associated with inflammation suppression, this decrease could translate into a heightened inflammatory response, modulating CYP levels.

PGRMC1 is apparently involved in regulating cellular glucose and lipid homeostasis [191,208,209,210,211,212,213] and may increase plasma membrane levels of growth and hormone receptors and glucose transporters [214,215] by influencing lipid raft formation [191,216]. The composition of membranes and the presence of lipid rafts affects the behavior of membrane enzymes as well as their accessibility to the substrate [217]. This may potentially affect levels of influx and efflux transporters that mediate xenobiotic transport. PGRMC1 seems, therefore, to influence indirectly the intracellular concentrations of xenobiotics and their metabolites, as well as affect CYP activity.

*Pgrmc1* was identified in a six-gene expression signature, determined from a cDNA microarray database of liver samples from adult male mice treated with non-genotoxic carcinogens [218]. *Pgrmc1* expression is induced by dioxin during rat liver tumorigenesis [122], as well as in the human breast cancer cell line, MCF-7 [219], and in women chronically exposed to industrial chemical pollutants [220]. Dioxin activates AhR, a trans-factor responsible for CYP expression induction, as described above [1,199,200]. The yeast homolog Dap1 was shown to be required for resistance to the DNA methylating agent methyl methanesulfonate [221]. The expression of a heme-binding defective PGRMC1 mutant and the resulting increased susceptibility of breast cancer cells to chemotherapeutic agents evidences a role for holo-PGRMC1 in regulating susceptibility to DNA damaging agents, similarly to its yeast homolog Dap1 [147]. Tumor tissue-specific delivery of let-7i miRNA, which downregulates the expression of PGRMC1 [222,223] and CYP19A1 [224], reversed paclitaxel-induced chemoresistance [225]. PGRMC1 contributes to doxorubicin resistance by promoting tumor cell viability, proliferation, and epithelial-mesenchymal transition (EMT) induction [226,227,228]. Glycyrrhizin and derivatives bind to heme-dimerized PGRMC1 and enhance erlotinib and cisplatin-induced cell death, also inhibiting the interaction of PGRMC1 with low-density lipoprotein receptor (LDLR), suppressing intracellular LDL uptake [229]. As such, PGRMC1 seems to be linked to modulatory effects in the metabolism of genotoxic compounds via interactions with xenobiotic-metabolizing CYP-isoforms, and of nongenotoxic compounds via interaction with non-xenobiotic CYP-isoforms (involved in steroids, fatty acids, cholesterol, and eicosanoids synthesis) or non-CYP proteins.

PGRMC1 has also been reported to play a role in cellular iron homeostasis (reviewed by Nguyen et al., 2023) [230] by (i) interacting with and regulating the terminal enzyme for heme biosynthesis, ferrochelatase, thus controlling heme release [131], (ii) upregulating hepcidin expression, a peptide hormone produced by hepatocytes that plays a role in iron homeostasis [231], and (iii) being able to donate heme to apo-cytochrome *b*_5_ (demonstrated in vitro) [131]. A recent study [232] supported previous implications of PGRMC1 in the cellular dynamics of Ca^2+^ [233,234,235,236,237] by identifying a specific interaction with the endosomal two-pore channel (TPC1), shedding light on its role in regulating ER-endosomal coupling. PGRMC1 at endosomal-ER membrane contact sites may support heme transfer [126,131] by clustering heme-binding proteins, mediating heme shuttling to specific hemoproteins without elevating cytosolic levels of labile heme [232]. Moreover, translocation of PGRMC1 induced by Ca^2+^ depletion leads to interaction with plasma membrane calcium channels, which promotes cancer cell migration and metastasis [238]. These studies corroborate the early findings regarding the multiple subcellular locations reported for PGRMC1. In addition, several typically microsomal CYPs have been reported to have dual targeting, both for the ER and mitochondria [239]; hence, a mitochondrial location of PGRMC1 could imply its interaction with mitochondria-targeted xenobiotic-metabolizing CYPs.

Sensitization of paclitaxel-tolerant cells by PGRMC1 was suggested to occur via increased levels of free fatty acids, lipid droplets, and fatty acid oxidation, and to promote sensitivity to ferroptosis (cell death mechanism driven by iron-dependent phospholipid peroxidation), by increased lipophagy and microtubule detyrosination [240,241,242]. *Pgrmc1* KO mice presented a predisposition to steatohepatitis, non-alcoholic fatty liver disease (NAFLD), hepatocellular carcinoma, and increased ER stress in the liver [178,243]. The loss of PGRMC1 seems to reduce the protection of hepatic cells against liver damage by the upregulation of alcohol-degrading enzymes, increasing acetaldehyde production and ER stress [244]. Surprisingly, CYP2E1 expression was demonstrated to be downregulated in *Pgrmc1* KO mice [244], which supports the results obtained by the earlier McGuire et al. study and the role of PGRMC1 in CYP protein stabilization [162]. PGRMC1 was also reported to act as a size-selective cargo receptor to drive ER-phagocytotic clearance of mutant prohormones [245] and recruit misfolded proteins for this clearance [246]. A role in autophagy [247,248,249] may explain the posttranslational stabilization of hepatic CYP protein levels observed by McGuire et al. (2021), as selective ER phagocytosis contributes to regulating the homeostasis of hepatic ER contents and consequently the levels of CYP enzymes [250].

In summary, PGRMC1 might indirectly modulate CYP activity through its involvement in signaling pathways that affect CYP expression, by affecting membrane localization of CYPs and intracellular substrate availability and accessibility to the enzymatic active center, through regulation of iron homeostasis and heme availability, or by regulation of ER contents and CYP protein levels. Thus, an intersection between iron and heme metabolism, oxidative stress, inflammation, and phagocytotic clearance seems to be regulated by or regulate PGRMC1 activity, consequently leading to indirectly altered CYP activity.

## 5. Current Knowledge Gaps, Challenges, and Future Perspectives

Alterations in expression, function, or conformation of protein factors modulating CYP activity may be a new avenue for studies regarding idiopathic disorders with CYP enzyme activity alterations, as previously suggested [37]. Mutations and polymorphisms of CYPs and CPR have been studied for their impact on therapeutic response and for their contribution to genetic variability in terms of therapeutic response and detoxification or bioactivation of xenobiotics [11,12,13,18,42,43,81,251,252]. Studies by Hughes et al. (2007) and others were pivotal to the acknowledgment of PGRMC1 as a CYP activity modulator and suggest that missense mutations and/or altered expression of PGRMC1 may lead to differentiated forms of human diseases associated with deregulated CYP activity and contribute to interindividual variations in xenobiotic metabolism. Despite all the advances in PGRMC1 research during the last two decades, much is still ambiguous or unclear, indicating the need for more detailed investigation.

As described above, the oxidation status of PGRMC1’s heme moiety seems to play a role in its functions. The “natural” physiological redox partner of PGRMC1 remains to be identified. Doubts remain regarding PGRMC1’s interaction with CPR, and whether CPR may donate electrons to PGRMC1, in analogy with cyt *b*_5_. Although the binding of PGRMC1 to CPR and its inhibition has been reported in in vitro systems overexpressing CPR and PGRMC1 [134], this was not corroborated in cells expressing endogenous levels of CPR [137,162]. This contradiction leaves doubts on in vivo PGRMC1:CPR interactions, as the used in vitro system might not properly reflect the in vivo stoichiometry of both proteins. PGRMC1’s ability to reduce CYPs similarly to cyt *b*_5_ has been indicated as improbable due to the differences in heme-coordination [137,160]; thus, its effect on CYP activity may not involve direct electron transfer. Instead, it may be defined by allosteric modulation, without obligatory electron transfer, similarly as has been described for the effect of cyt *b*_5_ on some CYP isoforms [253].

PGRMC1 has a broad reported distribution in the cell and may participate in transferring small hydrophobic ligands between intracellular membrane compartments and transferring prosthetic groups to the apoprotein or even participating in protein shuttling across membranes [156]. The possible implication of PGRMC1 in heme-chaperoning [126,131] may attribute a role as a heme-donor to CYPs, an issue that remains unclear and requires further study. Moreover, CO generated endogenously through heme degradation by HO, an enzyme whose activity is strictly dependent on electron donation by CPR, may be a regulator of CYP activity. HO regulates cellular heme homeostasis, and its upregulation may impact the function of heme proteins, including both CYPs and PGRMC1. Remarkably, PGRMC1 was suggested as part of a protein signature that predicts tumor hypoxia [254] and was found to be abundantly expressed in the hypoxic zone surrounding necrotic tumor tissue [190]. This might indicate that its expression is activated during cellular stress or that PGRMC1 expression is upregulated to minimize the inhibition of PGRMC1 dimerization, triggered by the increased CO levels. HO might regulate PGRMC1 activity not only through heme degradation but also by blocking its dimerization through increased CO production. Therefore, HO and PGRMC1, through their respective roles in heme degradation and synthesis, may disrupt heme/CO equilibrium and modulate heme availability. Their deregulated activities can impact CYP activity by influencing the availability of its cofactor. The effect of PGRMC1 on CYP activity is isoform-specific, and more studies should be conducted to ascertain if this is substrate-specific as well, as binding affinities of CYPs for cyt *b*_5_ and for CPR have been reported to be affected by both the CYP isoform and substrate [13,16,17,18,108]. This isoform-specific effect may be interpreted by different modes of interaction of CYPs with PGRMC1. The substrate affinity was found to be altered for some CYPs when in the presence of PGRMC1, while other CYPs demonstrated altered turnover rates [137]. These differences may be due to several factors, including differential affinities for PGRMC1 among CYP isoforms, as exists for cyt *b*_5_, and the interaction between the two proteins may induce differential conformational changes that influence interaction and affinity with either the substrate and/or with CPR [42,255].

The interaction between PGRMC1 and CPR reported by Szczesna–Skorupa and Kemper [134] could be indicative that PGRMC1 might be a redox partner of CPR, although interacting more strongly with CYPs than with CPR. As such, besides the allosteric effect, PGRMC1 could be a putative electron donor to CYPs, in a similar way to that of cyt *b*_5_. Following the contradictory reports provided by McGuire et al. [162], which did not find binding of FLAG-tagged PGRMC1 to CPR, it should be noted that the stoichiometry of the different protein partners in the CYP enzyme complex may greatly differ. An additional issue of model systems used for PGRMC1 overexpression is the recapitulation of its cellular location, which may be cell-type specific. Another concern in CYP electron-transport reconstitution systems is the proportion of each protein factor, as plausible stoichiometries for proteins of the CYP enzymatic complex need to be assessed to better recapitulate in vivo circumstances. Additionally, a substantial number of studies made use of tagged proteins. It should be noted that results obtained with tagged proteins must be carefully interpreted, due to structural effects tags may have on protein conformation and function. There is growing evidence of the influence even of small tags on protein dynamics, biodistribution, and function [256,257,258,259].

Expression of CYPs is quite well studied, while posttranslational control of CYP activity of most isoforms remains to be fully understood. The hypothesis proposed by McGuire et al. that PGRMC1 may stabilize CYP by covering ubiquitination regions prompts more studies regarding the interaction of these two proteins and their PTM status. Data from a previous study also suggested a model in which PGRMC1 inhibits the degradation of ubiquitinated proteins, which did not exclude the possibility that PGRMC1 may also inhibit the ubiquitination machinery itself [247]. PTM of PGRMC1 has been reported to affect binding to protein partners and to influence many of its biological functions, associated as well with its intracellular distribution and function following progesterone binding and its effect on cellular metabolism [141]. However, PTM studies regarding PGRMC1’s effect on the interaction and modulation of CYPs are scarce. This is also the case for the effect of progesterone binding (or other ligands for that matter) on the interaction of PGRMC1 with CYPs, and whether progesterone-bound PGRMC1 retains the ability to bind to CYP. This may be especially relevant in stages of increased hormonal concentrations, such as pregnancy and certain cancers, as increased binding of PGRMC1 to hormone ligands may result in changes in CYP activity, translating into altered drug efficacy and lack of therapeutic outcomes. In addition, heme-mediated dimerization of PGRMC1 and membrane trafficking may be mutually exclusive functions that could potentially be reciprocally regulated by phosphorylation/dephosphorylation at Tyr113 [156,260]. Moreover, although a disulfide bond between the two Cys129 residues had been observed in the crystal of PGRMC1 [151], the mutants previously assessed (Cys129Ala and Cys129Ser) had shown no significantly diminished heme absorption and dimerization [130,151]. However, as highlighted by new structural insights, the impact of the disulfide bond on PGRMC1 dimerization in the absence of heme had not been investigated [261]. The recent study proposes intermolecular disulfide bond formation through the previously overlooked Cys129 as another route for PGRMC1 dimer formation. Overall, a better understanding of PGRMC1’s heme-dependent and heme-independent functions is needed.

PGRMC1 also appears to be involved in druggable crosstalk between classical and nonclassical hormone signaling as well as other molecular signaling pathways [192]. The increase in circulating proinflammatory cytokines elicits changes in liver gene expression profiles, downregulating many drug-metabolizing enzymes, although few are upregulated [43,85,182]. Hence, PGRMC1 may also indirectly impact CYP activity by affecting pathways that modulate CYP enzyme transcription, for example through its role in NF-κB mediated stimulation and the resultant production of proinflammatory cytokines [85,178].

The selection of CYP quantification endpoints should be carefully made and interpreted as the nonuniformity in mRNA and protein induction responses may prevent a full understanding of CYP modulation [262]. Preferably, mRNA, and protein but mainly enzymatic activity should be examined, as their individual assessments may not properly recapitulate PGRMC1’s effects [263]. Furthermore, there is an increasing concern that the frequently used PGRMC1 inhibitor AG-205 may affect more than PGRMC1 activity, as recent reports highlighted [264,265]. Its mode of action remains unclear and raises questions regarding its specificity for PGRMC1. It appears to affect genes involved in steroidogenesis and to inhibit the synthesis of galactosylceramide and sulfatide, independently of PGRMC1. Conclusions drawn from studies using AG-205 as a PGRMC1 inhibitor may therefore need reinterpretation of data and reconsideration.

## 6. Final Remarks

As a final remark and considering future perspectives, the determination of the amino acid residues relevant for interaction with different CYP isoforms would clarify their role in this PPI, and molecular modeling might assist in understanding whether PGRMC1 interferes with substrate-enzyme binding and/or affects CYP enzymatic turnover. Also, the effect on CYP activity of identified PGRMC1 variants, such as the His165Arg mutant associated with premature ovarian failure [164], was not assessed for consequences regarding xenobiotic metabolism. The role of naturally occurring PGRMC1 variants in drug response variability may be of interest and should be studied in more detail. Besides the report that PGRMC1 might be involved in a protection mechanism against acetaminophen-induced liver injury described above [162], a gap remains in studies assessing the direct effect of PGRMC1 in in vivo pharmacokinetics.

**Figure 4:**
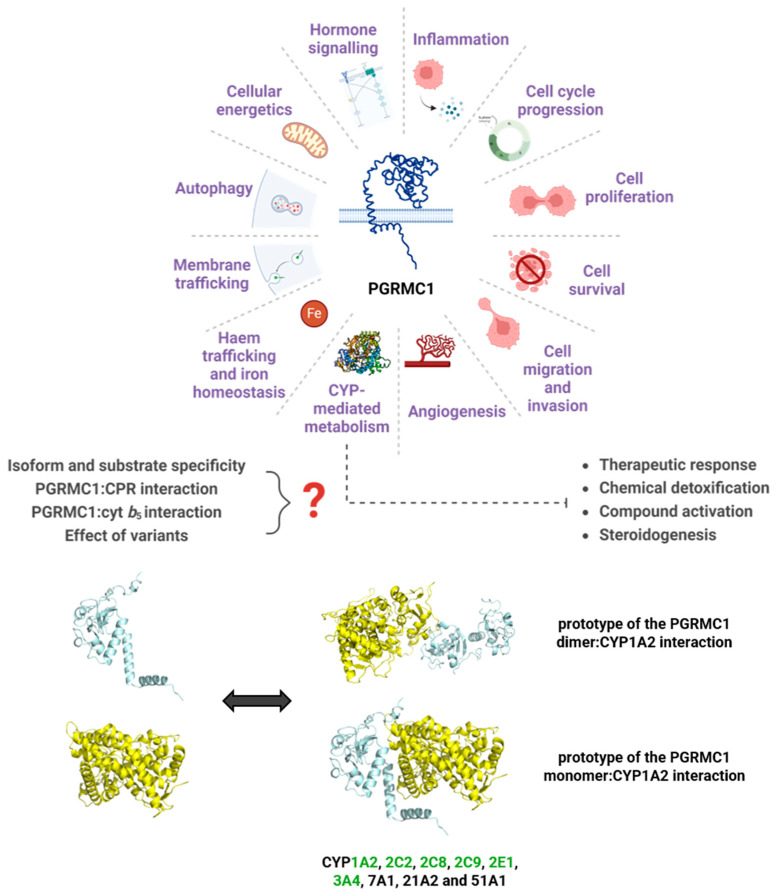
A myriad of pathways for which PGRMC1 has reported involvement, focusing on the relevance of a role in CYP-mediated metabolism. Represented are the prototypes for the interaction between PGRMC1 (in blue) and CYP1A2 (in yellow), predicted using the protein docking online tools ClusPro and HDOCK [269,270]. The ClusPro model was based on the crystal structures of the PGRMC1 dimer (PDB 4 × 8Y) and of CYP1A2 (PDB 2HI4), while HDOCK predicted the interaction model with the PGRMC1 monomer. Human CYP isoforms reported to interact with PGRMC1 [130,134,137,151,160] are noted, and highlighted in green are the CYPs belonging to families majorly involved in drug metabolism. Figure was created with BioRender.com, proteins are not to scale.

Several reviews have been published, describing the many roles and pleiotropic effects of PGRMC1, covering different research interests. Still, a large prominence of these reviews concerns oncology studies, particularly regarding PGRMC1’s role in ovarian, endometrial, breast, and lung cancers [30,32,117,266,267,268]. The divergent roles of PGRMC1 attributed since its discovery of evidence for its pleiotropic nature (Figure 4), from being credited as a CYP expression- and activity-modulator, associated with drug resistance, to its involvement in distinct pathways such as hormone signaling, heme, glucose, and cholesterol and lipid homeostasis, membrane trafficking, inflammation, and cell death regulation. This review aimed to summarize the most relevant findings on PGRMC1, focusing on its involvement in drug metabolism and steroid synthesis, particularly through its interaction with CYP-complex enzymes, considering the pleiotropic effects of this enigmatic protein. It also intended to highlight the importance of more extensive research as new and sometimes contradictory data appears at an increasing rate.

## Data Availability

Not applicable.
